# Organization of Community Mental Health Services for Persons with a Severe Mental Illness and Comorbid Somatic Conditions: A Systematic Review on Somatic Outcomes and Health Related Quality of Life

**DOI:** 10.3390/ijerph18020462

**Published:** 2021-01-08

**Authors:** Nicolaas Martens, Marianne Destoop, Geert Dom

**Affiliations:** 1 Collaborative Antwerp Psychiatric Research Institute (CAPRI), Antwerp University, B-2000 Antwerp, Belgium; marianne.destoop@uantwerpen.be (M.D.); geert.dom@uantwerpen.be (G.D.); 2 Multiversum Mental Health Services, B-2530 Boechout, Belgium; 3 Department of Health and Welfare, Karel De Grote University College, B-2018 Antwerp, Belgium

**Keywords:** community mental healthcare, severe mental illness, physical health, mental health nursing, collaborative care, public mental health

## Abstract

It is well established that persons with a severe mental illness (SMI) have a greater risk of physical comorbid conditions and premature mortality. Most studies in the field of community mental health care (CMHC) have only focused on improving cardiovascular health in people with a SMI using lifestyle approaches. Studies using organizational modifications are rather scarce. This systematic review aimed to synthesize and describe possible organizational strategies to improve physical health for persons with a SMI in CMHC. The primary outcome was Health-related Quality of Life (HR-QOL). Results suggested modest effects on quality of life and were inconsistent throughout all the included studies. Despite these findings, it appears that a more integrated approach had a positive effect on health outcomes, patient satisfaction and HR-QOL. The complexity of the processes involved in community care delivery makes it difficult to compare different models and organizational approaches. Mental health nurses were identified as possible key professionals in care organization, but no clear description of their role was found. This review could provide new insights into contributing factors for integrated care. Future research targeting the identification of the nurses’ role and facilitating factors in integrated care, in order to improve treatment and follow-up of somatic comorbidities, is recommended.

## 1. Introduction

Premature mortality in persons with a severe mental illness (SMI), such as schizophrenia, bipolar disorder or schizoaffective disorder, is high. Due to increased comorbidity with chronic medical conditions such as obesity, diabetes and cardiovascular impairment, it is now well established that persons with a SMI have on average a decline in life expectancy of between 15 and 20 years of age [1,2,3,4,5,6].

Presently, because of mental healthcare reforms worldwide, there is an increasing shift of residential mental health services into community mental health services, which is in line with the 2020 Mental Health Act of the World Health Organization. According to this health act the importance of an integrated health approach, targeting both mental and physical health, is pivotal [7].

However, a major problem in existing evidence targeting medical health problems in persons with a SMI is that most studies focus on lifestyle interventions as a way of optimizing physical health in persons with a SMI [8,9,10]. It can be questioned whether this is effective as a recent extensive meta-analysis on the effect of lifestyle interventions concluded that results were not clinically relevant in reducing cardiovascular risk factors [11]. In addition, evidence supporting the efficacy and effectiveness of existing guidelines is limited and the importance of social client-related factors and the complexity of real world settings should not be neglected in successful treatment of physical health in persons with SMI [12,13].

Whilst some research has been carried out on interventions focusing on physical health in people with SMI, to our knowledge no recent review has systematically studied the effect of specific modifications to healthcare organization, models of collaborative care and care coordination for persons with a SMI in a community mental health setting (CMHS), except for a Cochrane systematic review published in 2013 to assess the effects solely of collaborative care for persons with a SMI, primarily focusing on hospital admissions and quality of life as outcomes [14].

We conducted a systematic review of the evidence answering the following research questions: (1) which modifications in health care organization (e.g., care coordination, collaborative care approaches) in a CMHS are effective in improving the health-related quality of care & biometric outcomes (weight; Body Mass Index (BMI), lipid profile, glucose and blood pressure)? and (2) who are the specific stakeholders in these organizational interventions (disciplines and tasks)?

## 2. Materials and Methods

### 2.1. Data Sources

A systematic search was conducted between November 2019 and January 2020, using two electronic databases (Pubmed, Web of Science). Both searches made use of the same search strategy. The systematic review for all relevant literature was conducted using the PRISMA framework for systematic reviews [15,16].

The search strategy included the electronic databases Pubmed and Web of Science, using the following search string:

(“Organisation” OR “Organization” OR “coordination” OR “Coordinator” OR “coord *” OR “management” OR “Manager” OR “navigator” OR navigat * OR”collaborative care” OR “community mental *”) AND (“SMI” OR “serious mental illness” OR “severe mental illness” OR”seriously mental ill” OR “severely mentally” OR “affective disorder” OR “schizophrenia“ (MeSH Terms) OR “schizophrenic” OR “bipolar” OR “schizoaffective” OR “bipolar disorder”(MeSH Terms) OR “psychotic disorders“ (MeSH Terms) OR “schizophrenia” OR “bipolar” OR “psychosis” OR “psychotic”) AND (“physical” OR “somatic” OR “diabet *” OR “cardiovascular” OR “Metabol *” OR “hypertension” OR “weight” OR “Obesity” OR “body mass index” OR “BMI” OR “overweight”).

### 2.2. Study Selection

#### 2.2.1. Limits, Inclusion and Exclusion Criteria

To be included after a conscientious database search, studies needed to fulfill the following limits, inclusion and exclusion criteria. Limits: The search strategy was limited to articles published in English or Dutch between November 2009 and March 2020. Studies were considered eligible if the following inclusion criteria were met: (a) the intervention at hand should target somatic conditions in persons with a SMI living at home and/or under treatment at a CMHS; (b) the intervention consisted of an organizational modification (e.g., care coordination; care management; collaborative care; collocation or integration of services) in screening/assessment, follow-up or communication concerning comorbid somatic conditions; (c) the study was interventional (e.g., Randomized Controlled Trial; Controlled Trial; pseudo-experimental design). Interventional studies are found to be more accurate in assessing the direct impact of an intervention and less susceptible to bias than observational or qualitative study designs [17]; (d) the study reported quantitative data on (health-related) quality of life and/or somatic outcomes (weight, blood pressure, blood values); (d) persons with SMI included in the study were between 18 and 65 years of age. Studies were excluded if: (a) persons with SMI were receiving care from day-care or inpatient services or were homeless; (c) studies only evaluating the effect of an intervention targeting lifestyle modification without any changes in organization of the delivered healthcare.

#### 2.2.2. Quality Assessment

A quality assessment was completed using the CONSORT and ROBINS-1 appraisal tools to determine the methodological rigor and risk of bias. Both the CONSORT and ROBINS-1 tool are recommended by the Cochrane Collaboration in the appraisal of interventional studies [15,16,18].

### 2.3. Data-Analysis and Synthesis

Data was extracted according to the search strategy, the time frame of the search, interventions, selected outcomes, populations and the study setting. Data-analysis was performed by description of study characteristics, interventions and outcomes. No quantitative data analysis or data-pooling was performed. Results were reviewed by the co-authors to improve correct interpretation, relevance and comprehensiveness of the findings [19,20].

## 3. Results

**Figure 1:**
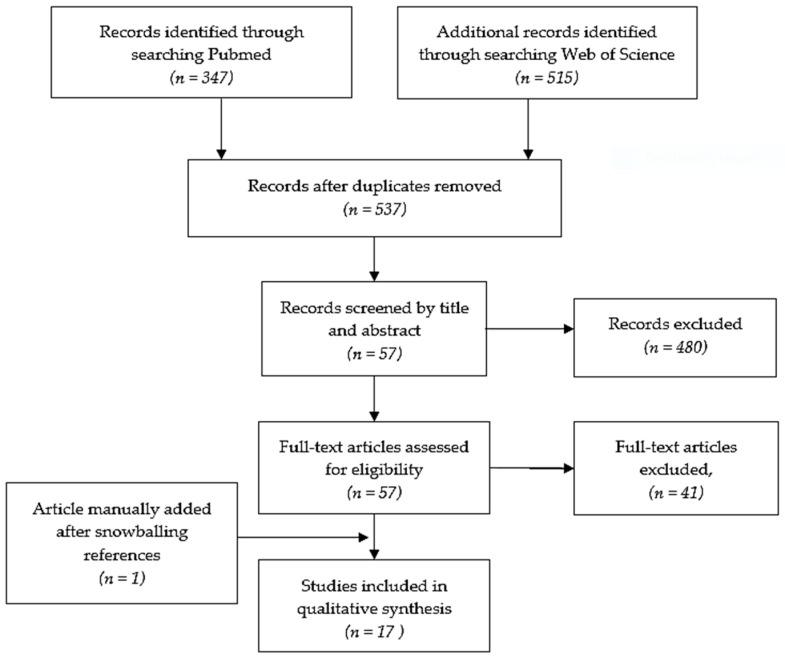
Search strategy Prisma Flow Chart.

The systematic literature search produced 3173 results in the database Pubmed. A total of 17 articles were included in this review [21,22,23,24,25,26,27,28,29,30,31,32,33,34,35,36,37]. After following the restriction criteria on age, language and type of research, results were limited to 347. After consulting titles and abstracts this was limited to 45 results. and after reading full text articles 13 results were withheld in this review [21,22,23,24,25,26,27,28,29,31,33,34,36]. An identical search string was used searching the database Web of Science, initially resulting in 3960 results. After setting limits for age, language and research type results were narrowed down to 515 results. After removing duplicates (*n* = 325) and after reading titles and abstracts (*n* = 178) the search resulted in 12 articles that were eligible for inclusion. After reading full texts, three articles were added to the review [30,32,35]. By means of snowballing one more article was manually added [37] because of partial results described in an included article [26]. The flow chart illustrating the strategy is shown in Figure 1.

### 3.1. Quality Assessment

A quality assessment was performed to identify differences in the trustworthiness of the results. As recommended by CHOCRANE, the CONSORT checklist was used for assessing interventional studies [38]. For the non-randomized studies, the ROBINS-1 Assessment tool was used [39]. Assessment of risks were divided into five domains (Participants selection; Intervention; Identifying confounders; Measurement; and Total Risk of Bias). Results are shown in Table 1. Three of the included studies showed a serious risk of bias, mostly contributed by the selection of participants, the low number in sample size (including one pilot study) [28] and the lack of identifying possible confounding factors influencing possible outcomes [24,29]. The latter can be contributed to the design of the intervention being pseudo-experimental without the use of controls [40,41,42]. Three studies showed a low risk of bias due to the use of an extensively described randomized design, a large sample size and the use of a clearly described intervention, including analysis of possible confounders [26,27,30,37]. All other studies showed a moderate risk of bias that can be attributed to a variety of reasons. It needs to be mentioned that no study showed a low risk of possible confounding factors, which can be caused by the complexity of community-based contexts that cannot be replaced by a controlled environment. An overview of the risk of bias is presented in Table 1.

A detailed overview of study characteristics can be consulted in Table 2. The included studies could be sorted into three different categories in line with the intervention sued in the studies: (1) implementation of organizational models of care; (2) formal training of health care workers; and (3) the application of educational/coaching interventions. A comprehensive overview of outcomes (quality of life and biometric outcomes) targeted in this review for each category can be consulted in Table 3.

### 3.2. Analysis

#### 3.2.1. Implementation of Organizational Models of Care

Van der Voort et al. [25] evaluated the effect of collaborative care for patients with a bipolar disorder on length and severity of symptoms of mania and depression. The intervention consisted of structured teamwork: a multidisciplinary team formulated a binding care plan, psycho-education, problem solving treatment, mood charting, mapping early warning signs, follow-up of psychopharmacological treatment and adherence. After 12-months the intervention group showed a significant decrease in number of months with depressive symptoms (Life Chart Method, z = 73.1, *p* = 0.002, d = 0.7) and severity of symptoms (Quick Inventory for Depressive Symptomatology, z = 72.9, *p* = 0.004, d = 0.4). No significant effects were detected on medication adherence and length or severity of manic symptoms.

Rogers et al. [35] evaluated the effect of a healthcare access model for persons with SMI that coordinated the interaction of primary care with mental health care. Patients regularly met with a nurse practitioner who coordinated care for each patient, implemented care by using existing or adding primary caregivers, communicated psychiatric symptoms with mental health centers, promoted lifestyle and improved accessibility to specialized healthcare. Some patients already had a nurse practitioner prior to the intervention, so a second control group, named the medium intervention, was added. The intervention group showed significant improvement in access to primary care (F = 3.56, df = 3.412, *p* = 0.01), comprehensiveness of care (F = 3.42, df = 3.419, *p* = 0.02) and community orientation of the provider (F = 4.00, df = 3.387, *p* = 0.008) compared to controls. The intervention group showed significant improvement in subscales ‘coordination of information’ and ‘utilization’ of the Primary Care Assessment Tool (F = 2.64, df = 3.414, *p* = 0.05).

A study evaluated the effect of a behavioral health home, located at a CMHS, providing care for cardiometabolic risk factors and comorbid conditions. Using weekly supervision meetings, care was delivered by a part-time nurse practitioner with prescribing authority and a full-time nurse care manager (provision of health education, supporting patients in attending appointments). Other caregivers made weekly rounds in the CMHS to improve integration of services. The intervention group showed significant differences compared to the TAU group in different domains: Proportion of indicated services received, diabetes care, prevention services in the intervention group, and primary care visits all increased significantly (*p* < 0.001; group-time interaction). Although the SF-36 showed a significant improvement in both mental (*p* < 0.001) and physical components (*p* = 0.003) in the intervention group, this was not significant in comparison with the control groups [31].

Gutierrez-Rojas et al. [34] determined the effectivity of basic screening for cardiovascular risk and metabolic syndrome (MS) combined with counselling: patients had four contacts over a period of 12-months’ Follow-up contacts included blood samples, weight, PANSS and Blood Pressure. Significant results were found in blood sample values. Symptoms assessed by the PANSS score also decreased significantly (80.7 (SD 25.4) vs. 69.7 (SD 24.9); *p* < 0.001). Regarding cardiovascular risk, a significant decrease in Framingham Risk Score was observed (8.4 (95% CI = 0.4–9.41) vs. 7.8 (95% CI = 6.93–8.75); *p* = 0.0353).

Druss et al. [30] implemented a manualized Care Management protocol where two full-time registered nurses delivered structured care to overcome patient, provider, and system-level barriers to primary medical care experienced by persons with mental disorders. The care managers served as a liaison with specialty medical and mental health providers and were notified about changes in the patient’s medication regimen and medical status. On the SF-36 scale, the intervention group showed improvement on the Mental Component (MCS), significantly higher than the control group (z = −3.15, *p* = 0.002). At 12-month follow-up, the intervention group had twice as many indicated physical examination activities (70.5% vs. 35.6%, (F1.361 = 166.83; *p* < 0.001), screening tests (50.4% vs. 21.6%, (F1.361 = 105.93, *p* < 0.001), more than four times as many educational interventions (80.0% vs. 18.9%, (F1.353 = 410.93, *p* < 0.001) and more than six times as many indicated vaccinations (24.7% vs. 3.8%, (F1.353 = 100.76, *p* < 0.001). The intervention group also had a significantly greater improvement in having a usual source of care (from 49.5% to 71.2%, versus 48.3% to 51.9% for usual care.

The ACTIVATE Mind and Body program was designed to raise awareness in the public mental health, primary care and non-governmental sectors about the physical and oral health care needs of people with SMI. The intervention included distribution of care guidelines for managing physical comorbidities to GP clinics and public mental health facilities and the development of a website for both health professionals and community members that provided mental health clinicians with the possibility of linking people with SMI to general practices The intervention group showed an increase in monthly rates of referrals for short, long and total GP consultations compared to the control group (*p* < 0.05) [29].

#### 3.2.2. Formal Training of Healthcare Workers

Eight of the included studies implemented an intervention that consisted of training healthcare professionals in providing care coordination and management. In the Primary Care Access, Referral, and Evaluation (PCARE) study of Druss et al. [30], two full-time nurses were trained to use a manual-based protocol in standardized care organization. At 12-month follow-up a significant increase in quality of primary care was found in the intervention group, including preventive medical care: physical examination activities (70.5% vs. 35.6% at baseline, *p* < 0.001), screening tests (50.4% vs. 21.6%, *p* < 0.001), educational interventions (80% vs. 18.9, *p* < 0.001), and vaccinations (24.7% vs. 3.8, *p* < 0.001). Compared to the control group, participants had an improved source of usual care. They also were more likely to visit the medical doctor, access to integrated cardiovascular care improved and more previously undiagnosed conditions were identified. A significant effect was also found on the mental health subscale of the SF-36 Quality of life scale (z = −3.15, *p* = 0.002).

The IMPaCT study assessed the effect of care coordination for persons with SMI in order to improve global health and substance use. Care coordinators in the intervention group were given a four-day IMPaCT training course based on the manualized implementation of IMPaCT. At 12-month and 15-month follow-up, no significant results were revealed in Quality of Life for both the mental and physical components. Of the secondary outcomes there was only a significant improvement in HDL cholesterol [32,43].

In the study of Kilbourne et al. [21], the Life Goals Collaborative Care framework was implemented by trained masters in patient education. 146 Participants were allocated to the Life Goals Intervention and 147 received TAU. The Life Goal intervention showed improvement in the physical component of the VR-12 questionnaire for Quality of Life (β = 3.21, *p* = 0.01, Cohen’s d = 0.39) and a decrease in LDL-values (β = −8.77, *p* = 0.04, Cohen’s d = −0.30). Kilbourne et al. (2013) also assessed the effect of the intervention on biometric somatic outcomes and physical health related quality of life (SF-12) in persons with a bipolar or schizo-affective disorder and a comorbid somatic condition. Participants in the intervention group showed a significant decrease of systolic (β = −3.1, *p* = 0.04, Cohen’s D = −0.22) and diastolic (β = −2.1, *p* = 0.04, Cohen’s D = −0.23) blood pressure compared to the control group. Although a significant decrease in manic symptoms (β =−23.9, *p* = 0.01) was observed in the intervention group, other outcomes did not differ significantly.

A study in Thailand implemented the Health Improvement Profile (HIP) developed by White et al. [44] and altered it to the context of Thailand (HIP-T). 10 community mental health nurses were trained in HIP-T using a three-hour workshop. At 12-month follow-up there was a significant decrease in BMI (Mean = 0.73/m^2^, *p* < 0.001; d = 0.2) and weight (Mean = 1.2 kg, *p* < 0.001; d = 0.1). Systolic blood pressure increased significantly (+2.47 mmHg, *p* = 0.003; d = 0.28). Results also showed a significant decrease in red flags on the questionnaire for BMI, pulse, foot care, sleep and breast checks [24].

Two related studies evaluated the effect of the CHANGE-trial respectively at 12 months and 24 months follow-up. The trail consisted of a comparison between lifestyle coaching, Care Coordination (both combined with TAU), and TAU. Both lifestyle coaching and care coordination were manually based interventions. Lifestyle coaching, for example, offered home visits to improve physical activity in daily life. Care coordination was provided by a trained psychiatric nurse to improve access to primary care. Analysis showed no significant effect on the Copenhagen mean age-standardized 10-year risk of CVD (8.4% (SD6.7%) in the CHANGE group vs. 8.5% (SD7.5%) in the care coordination group, and 8.0% (SD6.5%) in the treatment as usual group (*p* = 0.41). For secondary outcomes also no significant differences were found at 12-month follow-up. At 24 months follow-up, however, results were similar (10-year risk of CVD was 8.7% (SD 6.0%) in the CHANGE group vs. 7.7% (SD 5.7%) in the care coordination group, and 8.0% (SD 6.3%) in the treatment as usual group (*p* = 0.24) [26,43]).

Chwastiak et al. [28] pilot tested a CMHS-based collaborative care model derived from the primary care based TEAMcare model in ordeto treat type II diabetes among CMHC outpatients with psychosis. A combined team of a CMHS nurse care manager, a CMHC psychiatrist, an advanced practice registered nurse and an endocrinologist consultant received training in the TEAMcare model. Mean HbA1c among participants randomized to the intervention decreased from 9.4% to 8.3%, being both clinically and statistically significant (*p* = 0.049).

#### 3.2.3. Educational or Coaching Interventions

The Harp intervention, implemented by Druss et al. [23], was a peer led program improving self-management for somatic conditions in persons with SMI. Each session was led by two peer-specialists and was handbook based. Participants in the intervention group had a significantly higher improvement on the SF-36 scale. Significant secondary outcomes were the recovery assessment scale (0.15 points versus 0.08 points, *p* = 0.02) and activation scale (significant in case of time-interaction; F = 4.26, df = 2, *p* = 0.04).

Another intervention, named the “Living Well”, targets self-management tasks for various conditions. The intervention was delivered either by two mental health peers or a mental health provider and a peer co-leader. Outcomes showed, after a short period of significant decrease, no significant effects at follow-up on the SF-12 Scale. At 12-month follow-up, the intervention group had significantly higher mean scores on the 18-item Multidimensional Health Locus of Control (effect size = 0.66, *p* = 0.018) [33].

The “Bridge” intervention, studied by Kelly et al. [27], with improving self-management as its critical aim, used behavioral strategies as well as psychoeducation. Patients were supported by peer health navigators who each had caseloads of about 20 patients. Results showed higher quality relationships and more satisfaction with their primary care providers. The intervention group was significantly more likely to stay connected or become connected to primary care (80%) than those in the waitlist group (63%).

Sajatovic et al. [36] developed a Targeted Training in Illness Management (TTIM), targeting psychoeducation, problem identification, goal setting, behavioral modeling, and care linkage in combination with educational support by nurses, and social support and communication through peers. Psychiatric symptoms improved significantly in the intervention group versus TAU. There were no significant group differences in SF-36 scores or HbA1c values. However, diabetes knowledge improved significantly for TTIM versus treatment as usual (*p* < 0.001).

## 4. Discussion

The present study intends to determine the effect of organizational modifications on physical healthcare in persons with a SMI situated in Community Mental Health Care. Main outcomes of interest were primarily Health Related Quality of Life and biometric results. Secondly, the disciplines and tasks of persons involved in the included interventions were studied.

After analysis, the majority of the included studies (n = 14) implemented organizational modifications in order to improve the integration of physical healthcare and mental health services. Results could be divided into three categories: implementing a health care model that was innovative for the specific context, offering formal training, or offering coaching/educational services.

### 4.1. Formal Training

Most of the included studies organized manual-based training for health care providers. In terms of health-related quality of life, interventions that offered training in care coordination showed a modest effect on physical or health related quality of life or both. Interventions that offered a training program to health care workers about a model of care showed no clear effect on health-related quality of life. Effects of training on biometric outcomes were small, clinically not relevant and inconsistent over the different studies. One study that specifically targeted diabetes in people with SMI, however, showed clinically relevant results on levels of HbA1c [28].

These findings seem to be in contrast with previous research on training of health care workers in the general population, that shows a more direct link between training of community based health care professionals and somatic outcomes of patients involved [45,46]. Other studies, however, underline the scarce literature on how to implement a successful training module, whereby the context specific needs of the trainees should not be neglected. Integrating training in organizational policy, synthesis of possible barriers and facilitators, and an understanding of the trainees’ confidence and attitude should be assessed before the implementation of training. The literature also highlights the importance of continuous evaluation and adequate supervision during training [47,48,49,50]. Although training sessions described in the studies included in this review were mainly manual-based, only a limited description of the factors possibly influencing training effectiveness was provided, so specific conclusions concerning the effectivity of the training itself could not be made.

Some of the included studies aimed, after training caregivers, to evaluate interventions that considered Care Coordination as a stand-alone intervention, with modest results [28,32]. One study in this review, comparing care coordination in combination with lifestyle coaching, care as usual versus care coordination, and care as usual alone versus care as usual did not find significant differences in both physical and mental outcomes [26,37].

### 4.2. Implementation of Care Models

Of the six included studies that considered implementing a new model of health care delivery, outcome measures were mostly limited to biometric outcomes and access to health care services [25,29,30,31,34,35]. Five studies showed an increase in accessibility and number of contacts with relevant healthcare workers. The most obvious finding emerging from our analysis is that physical health outcomes are congruent with the degree in which services are integrated, with the most comprehensive way of integrating services being colocation, in which primary and mental health services are provided at the same location. The studies of Druss et al. [23,24] show a significant improvement in Quality of Life measured by the SF-36 and biometric outcomes such as Total cholesterol, blood pressure, HbA1C Framingham Risk Score and LDL. These findings are in line with studies on Integrated Care, where allocation of services is of great importance in improving accessibility, communication, continuity and quality of care, both in general health care settings and in CMHCs [51,52,53].

Three studies used the Framingham Risk score as an indicator for cardiovascular risk assessment and noted a significant decrease in cardiovascular risk [30,31,34]. Within the Framingham Risk sore, scores between 0 and 9.99 are considered a low risk, 10–19 medium risk and higher scores a high risk of having a future coronary event. Therefore, it can be questioned if the statistically significant decrease in the Framingham risk score as reported by these three studies can be considered clinically significant, because of a borderline medium risk or low risk score at both baseline and follow-up within the studies. No clear distinction was made between women and men, although gender is used in weighing the Framingham Risk Score [54,55,56].

Of the four studies that assessed Quality of Life as an outcome, three found a significant result on the applied (sub)-scales. Two studies found a significant increase in both the mental health and the physical health component of the SF-36, of which the improvement in the mental health component can be considered of clinical relevance [30,31]. This could be explained by previous research that found a link between physical activity and the reduction of symptom severity in persons with psychosis [57,58]. Another reason could be the fact that in both interventions, patients were assigned to a form of integrated healthcare, improving the accessibility of physical healthcare. The literature states that persons with a SMI experience multiple barriers in accessing appropriate physical healthcare, creating distress. Integrating services could eliminate barriers and improve mental wellbeing of persons with a SMI [2,59,60].

An important observation is the fact that many new models that are constructed and implemented are often based on the Chronic Care Model and the Rainbow Model, altered to mental health settings, but with modest effects on somatic outcomes. The literature, however, underlines the importance of rigorous implementation of these models [52,61]. Especially because of the complexity of community-based models, a more generic model of care could be of added value [62,63].

### 4.3. Educational and Coaching Interventions

The effects of educational or coaching interventions analyzed in this review are inconsistent in the Quality of Life and biometric outcomes. Most interesting is the finding that interventions that solely implement educational programs do not show a difference in outcomes compared to interventions that combine educational efforts with coaching, and other supportive contacts seem to have a more consistent effect on outcomes, such as Health Related Quality of Life and accessibility of primary care [23,27,36]. A recent systematic review that evaluated self-management approaches in persons with a SMI included the evaluation of educational efforts in improving Quality of Life and Symptomatic outcomes and reported only a small effect of educational efforts on the perceived outcomes [64]. Additionally, several studies emphasize the importance of social contacts in continuity of care, and contacts with peer coaches provides a solid base for trustworthiness and commitment in people with a SMI [65,66,67].

### 4.4. Disciplines, Roles and Tasks

As mentioned in the previous paragraph, peer-coaches or peer-led interventions are potentially valuable assets in improving physical health in persons with a SMI. Two recent systematic reviews emphasize the added value of peer-interventions in improving physical health in persons with a SMI. The most important outcomes were the improvement in self-management in health care and the support of peer-navigators. However, outcomes remained unclear and more research is needed to confirm these results [68,69].

In one study, nurses received training in and practiced the Health Improvement Profile [70], showing an increase in different health outcomes. Although the included study was at high risk of bias, the Hip method is a potential useful tool in planning care, and the study was recently reproduced in a Chinese context. However, results in a Western context were not to be found feasible in routine practice [44,71]. Interestingly, several studies mentioned the involvement of nurses in organizing and performing care. However, none of the studies clearly defines the tasks of the nurses involved in the interventions. Eight of the included studies did not formulate the presence or the role of a nurse practitioner in the intervention. More specialized nursing, such as a care manager role alongside conventional case management, could provide a more targeted monitoring of health needs such as metabolic symptoms [23,41,72,73,74,75]. In the case of the nursing role it is important to underline the fact that most of the applied models in the literature are based on the Chronic Care Model developed by Wagner [61], in which the nurse takes on a prominent role in providing holistic care, showing beneficial outcomes in depression and both physical and mental quality of life [76,77]. In the Guided Care Model also, which is based on the chronic care model, due to the transformation of mental health care into the community, the nursing role is highlighted as the key coordinating role to establish joint care planning between different primary care professionals [78]. A comparable European tool is the traffic light method in assessing health needs as suggested by Van Meijel et al. (2015) as a tool to improve health in persons with SMI [79].

Structured cooperation with GPs and keeping them and informed also seems to be crucial in achieving better health outcomes [25,29]. In terms of collaborative practices concerning persons with a SMI, integrated care models seem to lead to greater patient satisfaction, accessibility of care and quality of care. However, various barriers such as information exchange and funding mechanisms need to be addressed before successful implementation. It is also difficult to determine cost-effectiveness of integrated care models [51,80]. In addition, a recent article by The Lancet Psychiatry Commission (2019) regarding physical health of people with a mental illness also emphasizes the importance of installing integrated services handling both mental and physical health care, and the use of appropriate referral pathways [81]. The enhanced primary care pathway (EPCP), suggested by Röhricht et al. [82] is a possibility to formalize the collaboration between the general practitioner and secondary care services [82].

Although it was not described in the articles included in this literature review, the role of family carers could be of importance in improving physical health. Their role is often missed by research but can contribute to improvement in health status by assisting patients in their appointments and with their mobility. In addition, it should be mentioned that family carers often feel marginalized themselves by healthcare professionals, so their role needs greater acknowledgement [83,84].

Aside from professional roles and family carers, the quality of social relationships could also have an influence on physical health outcomes. For example, although not specifically addressing people with a SMI, research has shown the effect of social contacts on improvement of physical health and thus could implicate a topic of interest for future research [85].

Although our synthesis concludes in favor of a more integrated approach in improving physical health in persons with a SMI, the literature underlines the identification of a comprehensive model of integrated care to guide further development and evaluation of future interventions [86]. A focus on a more contextual research approach, as in the domain of implementation science, could provide a broader view of confounding and essential aspects in the implementation of interventions in community care. In the need for a more systematic strategy towards implementation, the PRACTIS framework as proposed by Koorts et al. could be an ideal framework in implementing community based interventions in a more systematic way with regard for the complexity of different contexts [13,80,87]. Evidence accentuates the possible use of digital tools in further research regarding physical health in mental healthcare [81].

From an economic perspective, the effect of organizational modifications, training or educational interventions on health economics remain unclear, as only one study reported a mean higher cost. In general, it is difficult to determine cost-effectiveness of integrated care models [29,51,81,88]. Future research should include economic data from both healthcare and other settings (labor, safety, justice) using the appropriate tools for evaluating either health economics or implementation costs [89,90].

Policy makers have a crucial role in promoting and facilitating physical health in persons with a SMI, because they are often perceived as a possible barrier or facilitator. Policy makers should invest in comprehensive, accessible healthcare for persons with a SMI, if possible in combination with easier access to housing/labor. At the macro-level, investments should be made in digital shared patient records, while policy at the meso- and/or micro-level should support the integration different services at the same location. The use of a universal and transparent framework could support policy makers in the development and funding of a more collaborative and integrated approach [81].

### 4.5. Limitations

This review has several limitations that need to be mentioned. First, most of the results showed a moderate risk of bias which makes it difficult and inconclusive to make any general assumptions about potential effectivity of the mentioned interventions. Secondly, because of the comparison of different interventions and the great variety in outcomes, it is difficult to interpret and compare, and results should only be seen as indicative. Third, it needs to be mentioned that collaborative or integrative care approaches are complex interventions with a variety of possible influencing factors, especially in community settings. Therefore, results need to be interpreted merely as guiding instead of as certain. Another result of this complexity is the impact on the level of evidence, which is rather low in the included studies.

## 5. Conclusions

This review examined possible strategies to improve physical health in persons with a severe mental illness in community settings. The literature shows modest effects on quality of life, but not consistently over different studies. No clear outcomes were shown, although it seems a more integrated approach has a positive effect on health outcomes and Health-related Quality of Care, and the use of peer-interventions could improve commitment to health services. Although, in most studies, nurses have a central role in care organization and delivery, their tasks and role concerning physical health in community mental health care can be an interesting topic for future research. The role of peers and the quality of social contacts should also not be overlooked. Overall, research targeting integrated care in the treatment and follow-up of somatic comorbidities, possibly using digital tools, is recommended. Authors should discuss the results and how they can be interpreted in the perspective of previous studies and of their working hypotheses. The findings and their implications should be discussed in the broadest context possible. Future research directions may also be highlighted.

## Figures and Tables

**Table 1:** Risk of Bias Assessment of the included studies.

Author	Risk of Bias Tool	Participants Selection	Intervention Bias	Identification Confounders	Measurement	Total risk of Bias
Cameron et al. [29]	ROBINS-I	Serious risk	Moderate risk	Moderate risk	Moderate risk	Serious
Chwastiak et al. [28]	CONSORT	Serious risk	Serious risk	Moderate risk	Low risk	Serious
Druss et al. [30]	CONSORT	Low risk	Low risk	Low risk	Low risk	Low
Druss et al. [31]	CONSORT	Low risk	Moderate risk	Moderate risk	Low risk	Moderate
Druss et al. [23]	CONSORT	Low risk	Moderate risk	Moderate risk	Low risk	Moderate
Gaughran et al. [32]	CONSORT	Low risk	Moderate risk	Moderate risk	Low risk	Moderate
Goldberg et al. [33]	CONSORT	Low risk	Low risk	Moderate risk	Low risk	Moderate
Gutiérrez-Rojas [34]	ROBINS-I	Moderate risk	Moderate risk	Moderate risk	Low risk	Moderate
Kelly et al. [27]	CONSORT	Low risk	Low risk	Low risk	Low risk	Low
Kilbourne et al. [22]	CONSORT	Low risk	Low risk	Moderate risk	Low risk	Moderate
Kilbourne et al. [21]	CONSORT	Low risk	Low risk	Moderate risk	Low risk	Moderate
Meepring, S. et al. [24]	ROBINS-I	Serious risk	Moderate risk	Low risk	Moderate risk	Serious
Rogers et al. [35]	CONSORT	Low risk	Moderate risk	Moderate risk	Low risk	Moderate
Sajatovic et al. [36]	CONSORT	Low Risk	Low risk	Moderate risk	Low risk	Moderate
Speyer et al. [37] Jakobsen et al. [26]	CONSORT	Low risk	Low risk	Low risk	Low risk	Low
van Der Voort et al. [25]	CONSORT	Moderate risk	Low risk	Moderate risk	Low risk	Moderate

Robins-I = risk of bias tool to assess non-randomized studies of interventions; CONSORT = Consolidated Standards of Reporting Trials.

**Table 2:** Overview of Included Studies.

Authors & Year	Study Design	Participants	Intervention:	Outcomes	Main Findings
Cameron et al. [29] Australia	non-randomized interventional study	(1) Adults with SMI who attended community based public mental health services for regular administration of either depot medication or clozapine.	The ACTIVATE Mind and Body program aims to raise awareness in the public mental health, primary care and non-governmental sectors about the physical and oral health care needs of people with SMI. The intervention included: (a) distribution of care guidelines for managing physical comorbidities to GP clinics and public mental health facilities; (b) the development of a website for both health professionals and community members; (c) development of strategies so that mental health clinicians actively linked people with SMI to general practices; comparison: TAU	Outcomes: (a) synthesis of GP consultations, coded in short visits (consultation <20 min) or long visits (consultation >20 min); (b) total cost to the patient and Federal Government; (c) data on pathology tests (cardiac enzymes or marker, electrolytes, full blood examination & coagulation, liver function tests, and urea, electrolytes, creatinine lipid studies and syphilis serology.)	(1) Outcomes: (a) increased short, long and total GP consultations than the control during the trial period (*p* < 0.05); (b) higher mean monthly costs to government for benefits paid for all GP claims during the trial period; (c) the intervention area showed a significant increase in the use of Liver Function Tests.
Chwastiak et al. [28] USA	RCT (Pilot Study)	(1) Adults aged 18 to 64 years old, diagnosis of schizophrenia, schizoaffective disorder, bipolar disorder, or major depressive disorder with psychosis. (2) Diagnosed with type II diabetes at least six months before enrollment; poor diabetes control (*n* = 35).	Pilot study of a CMHC-based collaborative care model based TEAMcare model for type II diabetes among CMHC outpatients with psychosis: (a) a team (CMHC nurse care manager, a CMHC psychiatrist, an advanced practice nurse) provided primary care onsite at the CMHC; (b) an endocrinologist consultant); (c) clinical visits and team meetings at the CMHC; (d) team trained in TEAMcare model; (e) health assessment and individualized health plan; (f) 30-min visits for 12 weeks; (f) diabetes education materials modified to target group; (g) nurses used behavioral interventions; and (h) coordinated care with primary and specialty care; comparison: TAU.	(1) Primary outcomes: (a) HbA1c after three months of intervention. (2) Secondary outcomes: (a) blood Pressure; (b) tobacco use; (c) mental health symptom measures; (d) process measures (nurse care manager visits).	(1) Primary outcomes: (a) at three-month follow-up, mean HbA1c among participants randomized to the intervention decreased from 9.4% to 8.3%, being both clinically and statistically significant (*p* = 0.049). In the TAU group, mean HbA1c decreased from 8.3% at baseline to 8.0% and was not significant. (2) Secondary outcomes: (a) both groups had statistically significant decreases in BMI (2.1 kg/m^2^ in intervention vs. 2.9 kg/m^2^ in TAU); (b) there were no significant changes in smoking or psychiatric symptoms.
Druss et al. [30] USA	RCT	(1) Adults with a severe mental illness. (2) In care at a Community Mental Health Center (*n* = 407).	(1) Care Management: (a) two full-time nurses used manualized protocol for care management; (b) provided information (medical conditions, available medical providers in the community, appointments); (c) at each meeting the client received an up-to-date booklet; (d) action plans to improve health behavior; (e) the care manager served as a liaison between medical and mental health providers; (f) coaching to help clients interact more effectively with their providers. (2) Duration: 12 Months; comparison: TAU, received contact information.	(1) Primary outcomes: (a) SF-36: Health-related quality of life; (b) quality of primary care (physical examination; screening tests; vaccinations and education; (c) Framingham Cardiovascular Risk Index.	Outcomes: (a) SF-36 improved on the Mental Component Summary score (MCS), (z = −3.15, *p* = 0.002); (b) quality of primary care showed twice as many indicated physical examination activities(70.5% vs. 35.6%, *p* < 0.001), more than double the screening tests (50.4% vs. 21.6%, *p* < 0.001), four times as many educational interventions (80.0% vs. 18.9%, *p* < 0.001), and six times as many indicated vaccinations (24.7% vs. 3.8%, *p* < 0.001); (c) the intervention also improved on having a usual source of care (from 49.5% to 71.2%, versus 48.3% to 51.9% for usual care(*p* = 0.001), and more primary care visits (4.94 vs. 4.11, *p* = 0.02); (d) a significantly greater number of undiagnosed medical conditions were identified by the intervention (11.9% vs.1.8%, *p* = 0.0046); (e) the Framingham Index was significantly lower at one year in the intervention group (6.9% vs. 9.8%, *p* = 0.03).
Druss et al. [31] USA	RCT	(1) Adults with a Serious mental illness. (2) Having a cardiometabolic risk factor: hypertension, hyperglycemic; cholesterol level >240 mg/dL and low-density lipo-protein (LDL) level >160 mg/dL (*n* = 447).	(1) The behavioral health home at a CMHS provided care for cardiometabolic risk factors and comorbid medical problems and included: (a) staff assignment (nurse with prescribing authority, nurse care manager); (b) weekly supervision meetings; (c) health education for lifestyle; (d) support patients to attend their medical appointments; (e) staff attended weekly rounds at the CMHC. (2) Duration: 12 months; comparison: TAU.	(1) Primary outcomes: (a) health care utilization and quality of care; (b) quality indicators for management of cardiometabolic risk factors. (2) Secondary outcomes: (a) blood samples (fasting blood levels of glucose; fractionated cholesterol, HbA1c); (b) Framingham risk score; (c) 36-item Short-Form Health Survey (SF-36); (d) patient activation.	(1) Primary outcomes: intervention showed significant differences compared to TAU on different domains: (a) the proportion of services received (67% to 81% vs. 65% to 63% in TAU; *p* < 0.001); (b) improvement of diabetes care (38% to 63% vs. 41% to 44% in TAU; *p* < 0.001); (c) quality of medication treatment (diabetic (81.0% vs. 67%; *p* = 0.04) and hypertension (92% vs. 75%; *p* < 0.01)); (d) improvement in prevention services (36% to 56% vs. 36% to 33% in TAU; *p* < 0.001); and (e) primary care visits increased compared to TAU (*p* < 0.001). (2) Secondary outcomes: (a) fasting blood levels of glucose; fractionated cholesterol and hemoglobin A1c (HbA1c); and (b) the SF-36 showed a significant improvement on both mental (29.9–38; *p* < 0.001) and physical component (40.5–42.9, *p* = 0.003) in the intervention group at 12 months follow-up but was not significant in comparison with the control group.
Druss et al. [23] USA	Group RCT	(1) Adults with a SMI. (2) Having a chronic general physical illness (*n* = 400).	(1) The Health and Recovery Peer intervention is a peer-led program for self-management: (a) a six-session program for persons with a SMI; (b) led by two certified peer specialists; (c) one-on-one peer coaching contacts were made; (d) four trained certified peer specialists functioned as interventionists. (2) Duration of the intervention: six months; comparison: TAU	(1) Primary outcomes: (a) Health Related Quality of Life (SF-36). (2) Secondary outcomes: (a) medication self-management; (b) medication self-assessment; (c) Patient Activation Measure; (d) assessment of diet; (e) Morisky Medication Adherence Scale; (f) Primary Care Contacts and (g) Recovery Assessment Scale (RAS).	(1) Primary outcomes: (a) SF-36: participants in the intervention group improved significantly on both the physical component and the mental health component compared to TAU (PCS: 2.7% vs. 1.4%; *p* = 0.46; d = 0.11); (MCS: 4.6% vs. 2.5%; *p* = 0.039; d = 0.17). (2) Secondary outcomes: significant differences between control and intervention group were found in: (a) RAS: At six-month (0.15% points in intervention group versus 0.08%, *p* = 0.02); (b) Patient Activation Measure: significant increase in the intervention group (+3.1 points vs. +1.5 in TAU, *p* = 0.01).
Gaughran et al. [32] UK	RCT	(1) Aged between 18 and 65 years with diagnosis of psychotic disorder.	An Integrated Health Promotion intervention: “IMPaCT therapy”: (a) implemented by patient’s usual caregivers after IMPACT-training and Physical Health awareness. (2) Duration: 9-months; comparison: TAU.	(1) Primary outcomes: (a) SF-36. (2) Secondary outcomes: (a) physical health measures; (b) substance use; (c) lifestyle measures diet, physical activity; (d) mental health status.	(1) SF-36: (a) no significant treatment effect for Physical or Mental health scores between TAU and IMPACT; (b) effects on physical health (d = −0.17 (12 months) and −0.09 (15 months)); mental health, (d= 0.03 (12 months) and −0.05 (15 months)). (2) Secondary outcomes: (a) significant difference in HDL cholesterol intervention; (b) reduction in waist circumference compared to control (−4.20 cm, *p* = 0.006).
Goldberg et al. [33] USA	RCT	(1) Adults with diagnosis of schizophrenia spectrum disorder or bipolar disorder with psychotic elements. (2) At least one chronic general medical condition (*n* = 63).	Goal of this study is to implement collaborative care to enhance patients’ self-management by structured collaborative practices: (a) a team that consists minimally of a nurse, patient, and psychiatrist, and possibly a family member; (b) treatment plan.	Primary outcomes: (a) SF-12 was used to assess global functioning and Quality of Life; (b) the 6-item Self-Management Self-Efficacy Scale: (c) the 13- item Patient Activation Scale; (d) the 18-item Multidimensional Health Locus of Control; (e) the 24-item Recovery Assessment Scale–Short Form; (f) 18-item Instrument to Measure Self-Management; and (g) the Morisky Medication Adherence Scale	(1) Primary outcomes: (a)The 12-item Short-Form Health Survey (SF-12), (b) Self-Management Self-Efficacy Scale, (c) the 13- item Patient Activation Scale showed statistically significant improvements in favor of the control group at post-intervention. At follow-up, however, none of these differences were significant.
Gutiérrez-Rojas et al. [34] Spain	Quasi–experimental	(1) Adults diagnosed with schizophrenia. (2) Being overweight (BMI+25). (3) Enrolled in community mental health center (n = 403).	(1) Determine the effectivity of basic screening for cardiovascular risk and Metabolic Syndrome (MS) combined with counselling: (a) for contacts over a period of 12-months; (b) during contacts participants were informed and information pamphlets were distributed.Comparison: baseline data at 12-month follow-up.	(1) Primary outcomes: (a) blood samples (cholesterol, glucose); (b) symptoms (PANSS); (c) waist circumference; (d) weight; (e) Framingham Cardiovascular Risk Score. (2) Secondary outcomes: (a) Global functioning: GAF-score; (b) Health related Quality of life: EQ-5D.	(1) Primary outcomes: Significant results were found for: (a) blood samples (blood glucose (mg/dL) (103.1 vs. 99.2, *p* = 0.0034); total cholesterol (mg/dL) (219.9 vs. 211.5, *p* < 0.0001); hdL Cholesterol (mg/dl) (47.5 vs. 49.5, *p* = 0.02); LdL Cholesterol (mg/dL) (139.7 vs. 132.9, *p* = 0.0023); triglycerides (mg/dL) (174.1 vs. 161.1, *p* = 0.0005)); (b) symptoms (PANSS score decreased significantly, (80.7 vs. 69.7; *p* < 0.001; (c) waist circumference (113.0 vs. 110.7, *p* < 0.0001) and (d) weight (93.4 vs. 91.4, *p* < 0.0001) decreased significantly; (e) the Framingham CVD risk score (vs. 7.8 *p* = 0.0353) also decreased. (2) Secondary outcomes: (a) GAF score showed significant improvement at the end of the study (52.7 vs. 60.3 *p* < 0.0001); (b) EQ-5D significantly increased (59.4 and 66.8, *p* < 0.001).
Kelly et al. [27] USA	RCT	(1) Adults diagnosed with schizophrenia, schizo-affective disorder, bipolar disorder, or major depression.	The “Bridge” intervention is: (a) a manualized intervention that uses motivational interviewing, cognitive behavioral strategies and psychoeducation; (b) is personalized to the healthcare experiences and needs of the participant; (c) aims to increase access, experience and self-management regarding healthcare; (d) facilitated by trained peer health navigators; (e) three peer health navigators had caseloads of about 20 each throughout the study. (2) Duration of intervention: 6-months; comparison: TAU.	(1) Outcomes: (a) the Working Alliance Inventory; (b) intervention fidelity and intensity; (c) health service utilization (‘UCLA CHIPTS healthcare’ and ‘health utilization survey’); (d) satisfaction with primary care provider (Engagement with the Healthcare Provider Scale); (e) self-management attitudes and behaviors; (f) checklist of 10 chronic health diagnoses; SF-12.	(1) Outcomes: (a) the intervention group reported higher quality relationships with their primary care providers; (b) intervention fidelity scores were above the “good” range; (c) health service utilization showed a trend for more routine health screenings in the treated group, and they also increased their visits to routine care providers more than the TAU group; (d) in Chi-square comparisons, the intervention group was significantly more likely to stay connected or become connected to primary care (80%) than those in the waitlist group (63%); (e) a checklist of 10 chronic health diagnoses: the intervention led to higher rates of diagnoses for the treatment group compared to the waitlist group; (f) significant reductions in the severity of bodily pain were reported by those in the treated group compared to the waitlisted TAU group.
Kilbourne et al. [22] USA	RCT	(1) Adults with diagnosis of bipolar disorder. (2) Presence of cardio-vascular risk factor; dyslipidemia; diabetes mellitus; obesity (BMI > 30); diagnosis of arteriosclerotic CVD (*n* = 118).	(1) Life Goals Collaborative Goals-program (LGCC): (a) self-management support; (b) care management: monthly follow-up, medical support; (c) guideline support to the caregivers involved. (2) Duration: 24 months; comparison: enhanced TAU.	(1) Primary outcomes: (a) physical health measures, (b) quality of life (SF-12). (2) Secondary outcomes: (a) HDL and LDL-levels; (b) BMI; (c) waist circumference; (d) Framingham Risk score; (e) additional outcomes.	(1) Primary outcomes: no significant effects for all primary outcomes (blood pressure; total cholesterol; SF-12). (2) Secondary outcomes: LGCC-arm showed reduced manic symptoms over the 24-month period (*p* = 0.01).
Kilbourne et al. [21] USA	RCT	(1) Adults with diagnosis of schizophrenia, bipolar disorder or major depressive disorder. (2) Presence of cardiovascular risk factor; dyslipidemia	(1) Life Goals Collaborative Care (LGCC): (a) five Self-management group sessions guided by trained educator; (b) Monthly Care Management calls to increase follow-up; (c) shared care-plans with caregivers. (2) Duration: 12 months; comparison: TAU.	(1) Primary outcomes: (a) quality of life (VR-12); (b) cardiovascular risk (blood pressure; BMI; physical activity (IPAQ-SF). (2) Secondary outcomes: (a) mental health symptoms; (b) Framingham Risk Score.	(1) In favor of the intervention there was a: (a) greater improvement on VR-12 physical health component scores (*p* = 0.01); significant reduction in low-density lipoprotein (LDL) levels (*p* = 0.04). (2) No significant difference in Framingham Risk Scores.
Meepring et al. [24] Thailand	Quasi–experimental	(1) Aged between 18 and 65 years, diagnosis of a SMI Exclusion: actively being treated for primary substance abuse (*n* = 105).	(1) The Health and Improvement Profile HIP was modified to the context of Thailand (HIP-T): (a) 10 Mental health Nurses were recruited; (b) the MHNs received a three-hour HIP-T training workshop. Comparison: baseline at 12-month follow-up.	(1) Primary outcomes: (a) BMI, (b) blood pressure. (2) Secondary outcome: (a) patient self-reported health behavior and potential health risks.	(1) Primary outcomes: (a) BMI showed a significant decrease (mean, 0.78/m^2^; *p* < 0.001); (b) significant decrease in weight (mean −1.13 kg; *p* < 0.001). (2) Secondary outcome: (a) results showed significant reductions in the number of participants with a red-flagged BMI at 12-months (*p* = 0.039) (pulse (*p* = 0.001), feet check (*p* = 0.004), sleep (*p* = 0.008), and self-checking of breasts (*p* = 0.002)); (b) a decrease of the total red flagged items for physical health pre and post-intervention (335, mean 3,19, (SD = 2.6) vs. 244, mean 2.32 (SD = 2.1), *p* < 0.001).
Rogers et al. [35] USA	RCT	(1) Adults having a serious mental illness. (2) Receiving mental health services as usual (*n* = 200).	(1) Min. three contacts with nurse practitioner (NP) who was situated at a CMHC: coordinated healthcare; complementary primary healthcare; address issues related to psychiatric condition; lifestyle, nutrition and exercise counseling; facilitate access to specialty care. (2) The NP followed guidelines including health assessment, diagnosis, and planning and was supervised by a physician in the community. Comparison: TAU with monthly educational sessions.	Outcomes: (a) SF-36 Quality of Life; (b) treatment outcome package (functioning, physical and mental health; (c) Multi-dimensional Health Locus of Control Form; (d) health beliefs; (e) The Primary Care Assessment Tool (PCAT) perceived quality of primary healthcare; (f) Nutrition, Prevention and Exercise Questions.	(1) Outcomes: (a) PCAT: Continuity of Care (*p* = 0.04) and the community orientation of the primary care provider (*p* = 0.05) showed significant differences favoring the intervention; (b) SF-36: social functioning increased more in the TAU-group compared to the High Exposure group (*p* = 0.01)
Sajatovic et al. [36] USA	RCT	(1) Adults with SMI (schizophrenia, schizo-affective disorder, bipolar disorder, major depressive disorder). (2) Comorbid type 2 diabetes (*n* = 200).	(1) Targeted Training in Illness Management (TTIM): (a) group-based psychosocial treatment; (b) educational support by nurses; and (c) social support and communication through peers. (2) Duration: 60 weeks; comparison: TAU	(1) Primary outcomes: (a) mental illness severity; (b) Global functioning (GAF); and (c) Health related Quality of Life (SF-36). (2) Secondary outcomes: (a) parameters linked to diabetes control (serum glycosylated hemoglobin, HbA1c); (b) knowledge of diabetes;	(1) Primary Outcomes: (a) psychiatric symptoms improved significantly at 60-week follow-up among TTIM versus treatment-as-usual participants; (b) improvement on GAF-score was significantly greater in the TTIM group vs. TAU; (c) SF-36 showed no significant group differencesHbA1c. (2) Secondary Outcomes: (a) HbA1c showed no significant differences, (b) diabetes knowledge improved significantly for TTIM versus treatment as usual (*p* < 0.001).
Speyer et al. [37] Jakobsen et al. [26] Denmark	Randomized, parallel-group Trial	(1) Adults with diagnosis of schizophrenia, schizoaffective disorder or persistent delusional disorder (ICD-10). (2) A waist circumference above 88 cm for women and 102 cm for men (*n* = 428).	Participants receive TAU alone or combined with lifestyle coaching or care coordination: (1) lifestyle coaching: (a) a manual-based intervention; (b) lifestyle coach offered home visits in daily life; (c) personal and professional networks were included; (d) patients could have contact with the team member for one year; (e) coach to participant ratio was 1:15. (2) Care coordination: (a) manual-based intervention; (b) a trained psychiatric nurse facilitated contact with primary care; (c) the coordinator to participant ratio was 1:40. (3) Duration of the study: 12-month follow-up (Speyer) and 24-month follow-up (Jakobsen). Comparison: TAU.	(1) Primary outcome: (a) Copenhagen Risk Score: the 10-year risk of cardiovascular disease. (2) Secondary outcomes: (a) cardio-respiratory fitness; (b) physical and lifestyle measures; (c) delf-reported physical activity (Physical Activity Scale); and (d) Quality of Life (MaNSA; EQ-5D).	Speyer (2016), at 12-month follow-up. (1) Primary outcomes: (a) The mean age-standardized 10-year risk of CVD was not significant. (2) Secondary outcomes: (a) no significant differences were found for any of the secondary outcomes. Jakobsen (2019), at 24 month follow-up. (1) Primary outcomes: the mean age-standardized 10-year risk of CVD showed a significant difference in sensitivity analyses of complete cases: the CVD risk was 9.0% (SD 5.9%) in the CHANGE group, 8.1% (SD 6.0%) in the care coordination group, and 7.8% (SD 6.1%) in the treatment as usual group (*p* = 0.08). (2) Secondary Outcomes: no significant differences were found for any of the secondary or exploratory outcomes.
Van Der Voort et al. [25] The Netherlands	Controlled trial	(1) Aged 18–65 years, diagnosis of bipolar disorder.	(1) Goal is to enhance patients’ self-management by structured collaborative practices: (a) a team (nurse, patient, and psychiatrist) (b) formulates a treatment plan and (c) offers psycho-education, (d) problem solving therapy, (e) mood charting, (f) early warning signs and (g) psycho-pharmacological and somatic care. (2) Duration of the intervention: 12-months. Comparison: TAU.	(1) Primary outcomes: (a) Global functioning: FAST-test; (b) Quality of Life (WHO Qol Bref)	(1) Primary outcomes: (a) Global functioning increased more in the intervention group compared to TAU (d = 0.3, *p* = 0.001); (b) at sox-month follow-up autonomy increased significantly in the intervention group compared to control and increased even more at 12-month follow-up (d = 0.5); (c) No significant differences were found in global Quality of Life; however, in the intervention group there was a significant increase for the physical health component.

Abbreviations: TAU = Treatment as usual; RCT = Randomized controlled trial; HDL = High density lipids; PANNS = Positive and Negative Syndrome Scale; CVD = Cardiovascular disease; HIP = Health improvement profile).

**Table 3:** Summary of intervention outcomes concerning the implementation of organizational models of care.

Author	Weight (kg)	BMI	HbA1c (%)	Blood Glucose (mg/dL)	Cardiovascular Risk	LDL (mg/dL)	Number of Screening Visits	Total Cholesterol (mg/dL)	Systolic BP (mmHg)	Diastolic BP (mmHg)	QOL
Implementation of Organizational models of care											
Cameron et al. [21]	/	/	/	/	Screening cardiac enzymes: 1.1 vs. 0.7 per 100 inhabitants	/	/	/	/	/	/
Druss et al. [23]	/	/	/	/	Framingham CRS7.8 (5.7)–6.9 (5.3) *	/	/	/	/	/	SF-36: Mental component:36.4 (10.1)-39.3 (9.91) * Physical component:36.4 (11.7)-37.1 (11.5)
Druss et al. [24]	/	/	6.4 (2.2)– 6.5 (1.6) *	113.7 (40.0)–111.9 (41.2)	Framingham CRS 10.4 (8.3)–9.2 (7.8) *	122.9 (40.7)–112.2 (39.5) *	/	202.6 (44.2)–193.8 (43.8) *	137.0 (19.6)–132.1 (17.3) *	89.5 (13.2)–83.6 (10.9) *	SF-36: Mental component: 29.9 (13.5)–38.0 (14.3) * Physical component: 40.5 (11.9)–42.9 (12.2) *
Gutiérrez-Rojas et al. [28]	93.4 (17.9)–91.4 (18.1) *	32.8 (5.1)–32.1 (5.3) *	/	103.1 (26.5)–99.2 (21.0) *	Framingham CRS: 8.4 (95% CI = 7.4–9.41)-7.8 (95%CI = 6.93–8.75) *	139.7 (42.5)–132.9 (36.5) *	/	219.9 (47.5)–211.5 (42.1) *		78.8 (10.8)–79.2 (9.9)	
Rogers et al. [33]	/	/	/	/	/	/	/	/	/	/	SF-36: No significant findings
Van Der Voort et al. [37]	/	/	/	/	/	/	/	/	/	/	WHOQoLBref: Physical component: 54.4 (16.2)–56.5 (18.0) * Overall Score: 3.3 (1.0)–3.4 (0.8)
Formal training healthcare workers											
Druss et al. [23]	/	/	/	/	Framingham CRS 7.8 (5.7)–6.9 (5.3) *	/	/	/	/	/	SF-36: Mental component:36.4 (10.1)–39.3 (9.91) * Physical component:36.4 (11.7)–37.1 (11.5)
Chwastiak et al. [28]	/	(−1.0 (CI:95%: −1.8; −0.1)) *	(−1.10 (CI:95%: −2.20; −0.01)) *	/	/	(−19.4 (CI:95%:−55.20; −16.5))	/	/	(−1.10 (CI:95%:−14.3; −12.0))	/	/
Gaughran et al. [26]	/	30.63 (7.52)–30.04 (7.67)	(−0.32 (CI:95%; −1.49–0.86))	/	/	/	/	/	/	/	SF-36: Mental component: 42.8 (13.73)–42.3 (13.42) Physical component: 47.44 (11.32)–47.54 (11.14)
Kilbourne et al. [30]	/	32.0 (6.2)–31.3 (5.8)	/	/	Framingham CRS 12.4 (8.9)–11.5 (6.4)	103.8(30.4)–105.6(39.5)	/	176.9 (37.2)–178.9 (45.5)	131.8 (16.4)–127.2 (15.4) *	80.7 (11.4)–75.9 (10.4) *	SF-12: Mental component: 32.7(7.7)–34.9(7.5) Physical component: 35.9 (7.2)–36.8 (6.6)
Kilbourne et al. [31])	/	34.01 (6.74)–34.07 (6.98) *	/	/	Framingham CRS 12.6 (7.7)–12.8 (8.7)	112.24 (35.52)–107.71 (34.18) *	/	182.65 (42.32)–179.47 (41.70)	135.39 (14.36)–135.95 (17.23)	76.44 (8.74)–75.46 (9.05)	VR-12:Mental component: 35.42 (12.23)–38.13 (12.41) Physical component: 32.41 (10.59)–33.61 (11.36) *
Meepring et al. [32]	63.9 (11.9)-62.8 (10.3) *	22.8 (4.1)–22.0 (2.8) *	/	/	/	/	/	/	115.1 (13.5)–116.6 (13.1)	72.8 (8.5)–75.2 (9.0) *	/
Speyer et al. [35]	103.1 (23.8) vs. 102.9 (21.7)	33.9 (5.9) vs. 34.4 (6.3)	5.6 (3) vs. 5.4 (2.1) *	/	Copenhagen risk score: 8.4 (6.7) vs. 8.1 (6.5)	/	/	/	128.7 (13.9) vs. 129.1 (14.1)	/	EuroQOL 1.4 (0.3) vs. 1.3 (0.3)
Jakobsen et al. [36]	105.9 (22.2) vs. 104.9 (22.1)	35.6 (8.6) vs. 34.4 (8.6)	5.5 (2.4) vs. 5.4 (2.4)	/	Copenhagen risk score: 8.7 (6.0) vs. 8.0 (6.3)	/	/	/	129.1 (13.0) vs. 128.3 (13.4)	/	MANSA score: 4.8 (0.1) vs. 4.9 (0.1)
Educational or Coaching Interventions											
Druss et al. [25]	/	/	/	/	/	/	/	/	/	/	SF-36:Mental component: 32.05 (11.8)–36.64 (12.3) * Physical component: 32.73 (10.9)–35.42 (11.0)
Goldberg et al. [27]	/	/	/	/	/	/	/	/	/	/	SF-12: No significant changes at follow-up
Kelly et al. [27]	/	/	/	/	/	/	0.92 (0.92)–1.20 (0.99) *	/	/	/	/
Sajatovic et al. [34]	/	35.44 (8.0)–36.46 (8.6)	8.0 (2.2)–7.69 (1.9)	/	/	/	/	/	134.99 (20.7)–134.12 (20.7)	/	SF-36:Mental component: 37.17 (10.6)–42.05 (11.1)Physical component: 39.38 (10.1)–39.65 (11.1)

(“*” = *p* < 0.05); (“/”= results were not presented in this study) (Abbreviations: BMI = Body Mass Index; BP = Blood pressure; Framingham CRS = Framingham Coronary Risk Score; LDL = Low density Lipids; MANSA = Manchester Short Assessment of quality of life; QOL = Quality of life; VR-12 = Veterans RAND 12 Item Health Survey; WHOQoLBref = World Health Organisation Quality of Life -short version).

## Data Availability

Not applicable.

## Abbreviations

BMI	Body Mass Index
BP	Blood pressure
CMHC	Community mental health center
CMHS	Community mental health setting
CONSORT	CONsolidated Standards of Reporting Trials
EPCP	Enhanced primary care pathway
EQ-5D	EuroQol-5 Dimension Quality of life
FAST	Functioning assessment short test
Framingham CRS	Framingham Coronary Risk Score
GAF	Global assessment of functioning
GP	General practitioner
HbA1c	Glycated hemoglobin (A1c)
HIP	Health Improvement Profile
HR-QOL	Health related quality of life
IPAQ-SF	International Physical Activity Questionnaire Short Form
LDL	Low density Lipids
MANSA	Manchester Short Assessment of quality of life
PANSS	Positive and Negative Syndrome Scale
QOL	Quality of Life
RAS	Recovery assessment scale
ROBINS-I	Risk of Bias in Non-randomized Studies-of Interventions
SF-12	12-item short form survey
SF-36	36-item short form survey
SMI	Severe Mental Illness
TAU	Treatment as usual
TTIM	Targeted Training in Illness Management
VR-12	Veterans RAND 12 Item Health Survey
WHOQoLBref	World Health Organisation Quality of Life-short version
